# *Aerococcus urinae* and *Globicatella sanguinis* Persist in Polymicrobial Urethral Catheter Biofilms Examined in Longitudinal Profiles at the Proteomic Level

**DOI:** 10.1177/1178626419875089

**Published:** 2019-09-19

**Authors:** Yanbao Yu, Tamara Tsitrin, Shiferaw Bekele, Vishal Thovarai, Manolito G Torralba, Harinder Singh, Randall Wolcott, Sebastian N Doerfert, Maria V Sizova, Slava S Epstein, Rembert Pieper

**Affiliations:** 1 J. Craig Venter Institute, Rockville, MD, USA; 2 J. Craig Venter Institute, La Jolla, CA, USA; 3 Southwest Regional Wound Care Center, Lubbock, TX, USA; 4 Northeastern University, Boston, MA, USA

**Keywords:** *Aerococcus*, *Globicatella*, proteomics, urinary tract, catheter biofilm, host-pathogen interaction, infection

## Abstract

*Aerococcus urinae* (*Au*) and *Globicatella sanguinis* (*Gs*) are gram-positive bacteria belonging to the family Aerococcaceae and colonize the human immunocompromised and catheterized urinary tract. We identified both pathogens in polymicrobial urethral catheter biofilms (CBs) with a combination of 16S rDNA sequencing, proteomic analyses, and microbial cultures. Longitudinal sampling of biofilms from serially replaced catheters revealed that each species persisted in the urinary tract of a patient in cohabitation with 1 or more gram-negative uropathogens. The *Gs* and *Au* proteomes revealed active glycolytic, heterolactic fermentation, and peptide catabolic energy metabolism pathways in an anaerobic milieu. A few phosphotransferase system (PTS)–based sugar uptake and oligopeptide ABC transport systems were highly expressed, indicating adaptations to the supply of nutrients in urine and from exfoliating squamous epithelial and urothelial cells. Differences in the *Au* vs *Gs* metabolisms pertained to citrate lyase and utilization and storage of glycogen (evident only in *Gs* proteomes) and to the enzyme Xfp that degrades d-xylulose-5′-phosphate and the biosynthetic pathways for 2 protein cofactors, pyridoxal 6′-phosphate and 4′-phosphopantothenate (expressed only in *Au* proteomes). A predicted ZnuA-like transition metal ion uptake system was identified for *Gs* while *Au* expressed 2 LPXTG-anchored surface proteins, one of which had a predicted pilin D adhesion motif. While these proteins may contribute to fitness and virulence in the human host, it cannot be ruled out that *Au* and *Gs* fill a niche in polymicrobial biofilms without being the direct cause of injury in urothelial tissues.

## Introduction

The genus *Aerococcus* that was first described in 1953^1^ morphologically and biochemically resembles *Enterococci* and *Staphylococci. Aerococci* are gram-positive, facultatively anaerobic, α-hemolytic bacteria. Difficulties to grow *Aerococci* in vitro and identify the genus using conventional microbial culture techniques (fastidiousness) explain why their presence in human clinical samples has been historically overlooked.^2^ Species most frequently associated with human pathogenicity are *Aerococcus urinae* (*Au*) and *Aerococcus sanguinicola*.^2^ High-resolution microbial identification techniques, such as MALDI-TOF and 16S rDNA gene sequencing, support the notion that *Au* is a more common cause of urinary tract infection (UTI), endocarditis, bacteremia, and urosepsis than previously thought.^2-6^ A third *Aerococcus* species that causes nosocomial infections is *Aerococcus viridans*.^7,8^ Clinical *Aerococcus* strains are resistant to sulfonamides. While mostly sensitive to treatment with vancomycin, carbapenem, and penicillin drugs (which remain the primary antibiotic drug treatment choices), cases of resistance to penicillin and cephalosporin have emerged.^2^ The first complete *Au* genome analysis (strain ACS-120-V-Col10a) was deposited in Genbank in 2012 and archived in the European Nucleotide Archive.^9^ Additional closed *Aerococcus* genomes were published by Carkaci et al^10^ in 2016; this included *A. sanguinicola* CCUG 43001T, *Au* CCUG 36881T, and *A. viridans* CCUG 4311T. Recently, *Au* and *A. sanguinicola* genomes were characterized, postulating a core genome and determining the extent of genomic diversity among various clinical isolates.^11^ *Au* genome annotations suggest that the strains harbor between 1680 and 1880 coding genes. The *Au* genomes ranged from 1.9 to 2.4 MB, and the *A. sanguinicola* genomes from 2.01 to 2.12 MB in size. Putative virulence genes, based on sequence homology data, were identified for both species: the *Au* genes were orthologs to *htpB* (encoding a Legionella heat shock protein), *lap* (a Listeria adhesion protein), *lmb* (a *Streptococcus agalactiae* laminin-binding protein), *fbp54* (a *Streptococcus pyogenes* fibronectin-binding protein), and *ilpA* (a *Vibrio* immunogenic lipoprotein). A capsular polysaccharide (CPS) biosynthesis locus was also identified.^11^ Phylogenetic clustering suggests that an *Au* clade causing UTI, bacteremia, and endocarditis was distinct from other clades associated with the diagnosis of UTI or bacteremia.^11^ In one of a few functional studies relevant to infection, *Au* was reported to activate human platelets and form biofilms.^4^ Comprehensive proteomic analyses of *Au* isolates in either the planktonic or the biofilm milieu have not been reported to date.

The genus *Globicatella* is also a member of the family Aerococcaceae. The first cases of infection associated with *Globicatella sanguinis* (*Gs*) were reported in 1992.^12^ Like *Au*, the species is gram-positive, facultatively anaerobic, and α-hemolytic, and forms pinpoint colonies on blood agar plates under microaerophilic conditions.^13^ Similar to *Au*, more common infections attributed to this rare pathogen are UTI, meningitis, and bacteremia.^12^ Based on non-culture-based identification methods such as 16S rDNA analysis, human *Globicatella* isolates phylogenetically resemble the species *Globicatella sulfidifaciens*, which is not a human pathogen.^14^ *Gs* has morphological and genetic traits similar to *Aerococcus viridans*,^8^ and can be distinguished from *Streptococcus* and *Aerococcus* spp. by 16S rDNA and MALDI-TOF analysis and the sequence of *sodA*.^13,14^ *Gs* strains were reported to be resistant to macrolides, clindamycin, and cefotaxime.^13-15^ Whole shotgun genome sequence data were deposited in the European Nucleotide Archive for the strain *Globicatella* sp. HMSC072A10; the genome annotation lists 2027 open reading frames (ORFs) (taxonomy ID 1739315; GCA_001811625.1). The *Gs* strain UMB0514 (taxonomy ID 13076; GCA_002847845.1) was sequenced and is predicted to harbor 2029 ORFs. Comparative genomic analyses of clinical strains are not yet available. Among the common traits of *Gs* strains appear to be α-hemolysis and preferably aerobic growth. *Gs* strains are catalase- and pyrrolidonyl arylamidase-negative.^16^

*Au* and *Gs* infections have been associated with old age.^2,6,14,17^ In studies of polymicrobial colonization of indwelling urethral catheters, we identified *Au*^18^ and *Gs* (published here) as catheter biofilm (CB) cohabitants. To our knowledge, little is known as to how these bacteria adapt to low-nutrient, low-oxygen conditions at the catheter surface-urothelial tissue interface. Where to place the species on the urovirulence scale is uncertain although young, immunocompetent human hosts are rarely colonized. The goal of this proteomic investigation is to shed light on these questions. Via the analysis of metaproteomic data derived from clinical samples and in vitro cultured bacteria, we gained insights into the metabolisms of *Au* and *Gs* strains as cohabitants of polymicrobial biofilms, transport systems needed to acquire nutrients and stress responses in a host milieu characterized by chronic innate immune responses.

## Methods

### Ethical statement

A human subject protocol and consent form describing the risks of participation in the study were established by investigators at Southwest Regional Wound Care Center (SRWCC) in Lubbock, Texas, and the J. Craig Venter Institute (JCVI) in Rockville, Maryland. Approval under the study number #56-RW-022, reviewed by the Western Institutional Review Board (WIRB) in Olympia, Washington, and IRBs at the JCVI and Northeastern University (NEU) in Boston, Massachusetts, was obtained in 2013. Only adults were enrolled and provided written consent. Catheter specimens were collected firsthand for this study, as patients visited the clinic due to a medical need to replace the Foley catheters on a regular basis. Scientific staff analyzing the clinical specimens using culture-based, genomic, and proteomic methods at the JCVI and NEU did not have access to medical records with information allowing patient identification. The electronic and printed medical records generated at SRWCC were retained for 4 years to redact the records followed by the integration of relevant medical data into multi-omics analyses. Medical data at the clinical site were destroyed thereafter.

### Patient clinical backgrounds and specimens

The patients whose CB samples were examined were part of a parent study based on prospective sampling in which 9 subjects with spinal cord injuries and neurogenic bladders were enrolled. These patients used indwelling urethral catheters for bladder management on a permanent basis. Catheter bag-derived urine and catheter specimens as well as medical data were obtained from each subject over a 3- to 6-month time frame to examine the molecular microbial-host crosstalk. Most patients also suffered from chronic wounds which were treated during physician’s office visits. Routine care included catheter exchanges to minimize the risk of catheter-associated urinary tract infections (CAUTIs). The formation of crystalline or non-crystalline biofilms on catheters and urinary pH changes were also monitored. The genera *Aerococcus* and *Globicatella* were identified from multiple samples derived from 1 patient each (P5 and P6). Given our interest in rare pathogens, we focused on their in vivo metabolisms in the article.

### Catheter sample preparation for microbial cultures

The latex catheter specimens were cut into 1- to 1.5-inch pieces and processed to extract DNA and protein for metagenomic and metaproteomic analyses. Catheter pieces from P5 were also processed to isolate microorganisms residing on the abiotic surface without freezing. To allow the recovery of fastidious bacteria preferring anaerobic growth conditions, freshly collected catheter pieces were placed in a casamino acid-based media at pH 6 and flushed with nitrogen gas as previously described.^18^ In an anaerobic glove cabinet, biological materials dispersing from catheter surfaces were plated on trypticase-yeast (TY) extract agar containing sheep blood (25 mL/L).^18^ In addition to those experiments, P5 and P6 catheter pieces were stored in polypropylene tubes at −20°C, shipped to the JCVI, and stored frozen at −80°C until further use. To recover viable microorganisms from these biomaterials, catheter pieces submerged in phosphate-buffered saline (PBS) were scraped to dislocate cells present on catheter surfaces. These extracts were plated on 5% sheep blood agar and brain heart infusion (BHI) agar and then grown aerobically with or without 5% CO_2_ for 24 to 72 hours at 37°C.

### Liquid and blood agar cultures to recover bacteria for proteomic studies

Biofilm extracts of P5 were incubated for up to 10 days. Colonies were picked from plates with a sterile loop and re-inoculated into liquid TY media supplemented with 1% human serum, conducting all experimental steps in a glove box for anaerobic subcultures.^18^ Subcultures grown from a small bacterial colony resulted in identification of the genus *Aerococcus* by 16S rRNA sequencing. A glycerol stock was prepared by mixing 1 mL of 40% glycerol and the suspension culture. This stock served as the inoculum for an anaerobic culture in 10 mL liquid trypticase soy broth (TSB; #43592; Sigma-Aldrich, St. Louis, Missouri) without agitation overnight at 37°C. From a slow-growing culture, bacterial cells were collected via centrifugation at 3200*g* for 15 minutes at 20°C. The flash-frozen cell culture pellet (CCP) was shipped to JCVI. *Au* isolates were not obtained from catheter extracts that were frozen at −20°C immediately after collection with subsequent cultures in an aerobic milieu on 5% sheep blood agar or in BHI or TY broth. In contrast, a frozen aliquot from a P6 catheter piece (#53_CB), streaked out on 5% sheep blood agar and incubated with or without 5% CO_2_ at 37°C, revealed gray pinpoint colonies with an α-hemolytic zone following 48 hours of growth. The colony morphology and microscopic traits (Zeiss Axioscope, 100× magnification) matched those previously reported for *Globicatella*.^19^ The colonies did not grow in TY or BHI broth overnight. To recover sufficient biomass for proteomic experiments, a single blood agar colony of the *Gs* strain was re-plated on blood agar and incubated with 5% CO_2_ at 37°C for 48 hours. Bacterial cells were harvested, washed with PBS, and centrifuged at 3200*g* for 15 minutes at 20°C to obtain a CCP. This pellet was flash-frozen prior to further analysis.

### CB extraction for proteomic analyses

The catheter pieces and urine pellet (UP) samples derived from catheter collection bags were thawed and processed as reported previously.^18,20^ Briefly, a catheter piece was placed in a 15 mL Falcon tube with 2 to 3 mL CHO buffer (100 mM sodium acetate, 20 mM sodium meta-periodate, and 300 mM NaCl; pH 5.5). Sodium meta-periodate weakened bacterial cell walls by oxidizing cell wall carbohydrates. Carbohydrate oxidation (CHO) buffer-suspended catheter pieces were sonicated in a ultrasonic water bath for 10 minutes allowing the biofilms to detach from the latex surface and vortexed. Sonication and vortex steps were repeated. Prior to processing a UP sample, the urine pH was adjusted to ~6.5 to 7.5 with 1 M Tris-HCl (pH 8.1). Via centrifugation at 8000*g* for 15 minutes, supernatant (CB_sup_) and pellet (CB_pel_) fractions were recovered. The volume of the CB_sup_ fraction was reduced to ~0.5 mL using an Ultrafree-4 membrane filter (10 kDa MWCO) by centrifugation at 3200*g* and exchanged into PBS. The CB_pel_ fraction was not re-suspended. Fractions were stored at −80°C until further use for proteomic analyses.

### Preparation of CCP and clinical sample lysates for proteomics

All UP, CB_pel_, CB_sup_, and CCP samples were lysed with the SED solution (1% aqueous sodium dodecyl sulfate (SDS), 5 mM ethylenediaminetetraacetic acid [EDTA], and 50 mM dithiothreitol [DTT]) in low protein adsorption microcentrifuge tubes in a 1:5 volume ratio. Samples were sonicated in a Misonex 3000 ice bath sonicator (ten 30 seconds on/off cycles at amplitude 6.5), moved to a heat block (95°C) for 3 minutes, and incubated at ambient temperature to complete lysis with occasional vortex steps at 20°C for 15 minutes. Lysates were cleared by centrifugation at 13 100*g* for 10 minutes. Approximately 10 to 20 µL lysate aliquots were loaded on SDS-PAGE (sodium dodecyl sulfate-polyacrylamide gel electrophoresis) gels, and protein bands were visualized. The total protein concentration was estimated by gel-staining with Coomassie Brilliant Blue-G250 (CBB). A 2 µg bovine serum albumin (BSA) quantity served to estimate protein quantities in cleared lysates from the comparison of staining intensities. Aliquots of lysates containing 100 µg total protein were subjected to filter-aided sample preparation (FASP) in Vivacon membrane filters (10 kDa MWCO; Sartorius AG, Germany), and sequencing-grade trypsin was used to completely digest proteins as reported previously.^21^ Peptide mixtures were desalted using a modified Stage-Tip method,^22^ lyophilized, and then ready for liquid chromatography coupled to tandem mass spectrometry (LC-MS/MS) analysis.

### Shotgun proteomics using LC-MS/MS

Dried peptide mixtures were re-suspended in 10 μL 0.1% formic acid (solvent A). The LC-MS/MS workstation was composed of the LTQ-Velos Pro ion-trap mass spectrometer coupled to the Easy-nLC II system via a FLEX nano-electrospray ion source (Thermo Scientific, San Jose, California). Detailed LC-MS/MS analysis steps were previously described.^23^ The sample was loaded onto a C_18_ trap column (100 μm × 2 cm, 5 μm pore size, 120 Å) and separated on a PicoFrit C_18_ analytical column (75 μm × 15 cm, 3 μm pore size, 150 Å) at a flow rate of 200 nL/min. Starting with solvent A, a linear gradient from 10% to 30% solvent B (0.1% formic acid in acetonitrile) over 195 minutes was followed by a linear gradient from 30% to 80% solvent B over 20 minutes and re-equilibration with solvent A for 5 minutes. The column was washed thrice with a 30-minute solvent A to B linear gradient to minimize cross-contamination. Peptide ions were analyzed in an MS^1^ data-dependent mode to select ions for MS^2^ scans using the software application XCalibur v2.2 (Thermo Scientific). The ion fragmentation mode was collision-activated dissociation with a normalized collision energy of 35%, and dynamic exclusion was enabled. MS^2^ ion scans for the same MS^1^ *m/z* value were repeated once and then excluded from further analysis for 30 seconds. Survey (MS^1^) scans ranged from the *m/z* range of 380 to 1800 followed by MS^2^ scans for selected precursor ions. The 10 most intense peptide ions were fragmented in each cycle. Ions unassigned or having a charge of +1 were rejected from further analyses. Two technical LC-MS/MS replicates were run for each sample. Their raw MS files were combined for the database search steps.

### Computational methods to quantify the metaproteomes

Raw MS files from CB_sup_ and CB_pel_ fractions were merged prior to database searches. All raw MS files were searched using the Sequest HT algorithm integrated in the software tool Proteome Discoverer v1.4 (Thermo Scientific). Technical parameters and database construction were described previously.^21,24^ Only rank-1 peptides with a length of at least 7 amino acids were considered for analysis. False discovery rates (FDRs) were estimated using the Percolator tool in Proteome Discoverer v1.4 with a (reverse sequence) decoy database. Protein hits identified with a 1% FDR threshold were accepted, and the “protein grouping” function was enabled to ensure that only 1 protein was reported when multiple proteins shared a set of peptides. The initial database searches were performed using reviewed protein entries of a non-redundant human UniProt dataset (release June 2015; 20 195 sequences), and protein entries were derived from 23 microbial genomes known to colonize and infect the human urinary tract^20,24^ (Supplemental File S1). This included the *Au* strain ACS-120-V-Col10a (UniProt-ID UP000008129). Database searches were customized based on 16S rRNA genus IDs, including *Globicatella* sp. HMSC072A10 (UniProt-ID UP000176615) for MS files of P6 samples and other fastidious bacteria for MS files of P5 samples as reported.^18^ Further proteomic database search modifications had the goal to verify the databases yielding most *Au* and *Gs* protein matches: *Gs* strain UMB0514, *Globicatella sulfidifaciens* strain DSM 15739, *Aerococcus sanguinicola* strain CCUG43001, *Aerococcus christensenii* strain CCUG28831, and *Aerococcus viridans* strain ATCC 11563-CCUG 4311. All UniProt proteome and taxonomy IDs are included in Supplemental File S1. This iterative process included a reduction of the proteomic search space (eliminating species absent in the analyzed samples) and an optimization of strains (genotypes) of species of interest for this study. The process maximized correct and minimized incorrect assignments of indistinguishable peptides to proteins by the Proteome Discoverer software. For P5 datasets, the *Au* proteome (ID-UP000008129) had more than 90% of all peptide-spectral matches (PSMs) related to the genus *Aerococcus*. For P6 datasets, 60% and 40% of PSMs pertained to *Globicatella* sp. HMSC072A10 proteins and *Gs* strain UMB0514 proteins, respectively. This result suggests that the reference genomes represent genotypes similar to each other, and that a single *Gs* strain is identified in the CBs of P5. Therefore, we blasted .fasta sequence files for both proteomes and used a 90% sequence identity cutoff (Cd-Hit)^25^ to generate a non-redundant *Gs* hybrid protein sequence database harboring a total of 2384 protein sequences. Total PSMs per experiment were used to estimate normalized abundances of each individual protein (PSMi/∑PSM). PSMs summed for a given species (including human) served to determine contributions to the entire biomass in CB and UP samples.

### 16S rRNA analysis

The sample preparation and 16S rRNA sequencing and taxonomic profiling methods, using a MiSeq (Illumina) sequencing platform and UPARSE-based phylogenetic analysis, were identical to methods which we used previously.^18^ Unbiased, metadata-independent filtering was used at each level of the taxonomy by eliminating samples with less than 2000 reads.

### Protein functional and biological pathway analyses

The annotations of protein-encoding genes in the *Au* and *Gs* in silico reference proteomes are unreviewed. To our knowledge, neither species has any experimentally characterized proteins. To gain more insights into potential protein functions, we conducted sequence homology searches with BlastP in UniProt to identify bacterial orthologs from other species, particularly *Streptococcus*, with information on functional roles. Data from genes and proteins (and orthologs) were further analyzed using the databases Metacyc.org, Ecocyc.org, UniProt, and relevant literature. This allowed the interpretation of biological pathways, functions, and the identification of export signal sequences, cell wall localization motifs, and transmembrane domains.

## Results and Discussion

### Au and Gs recurrently colonize urethral catheter surfaces dominated by 1 or more gram-negative pathogens

**Figure 1.**
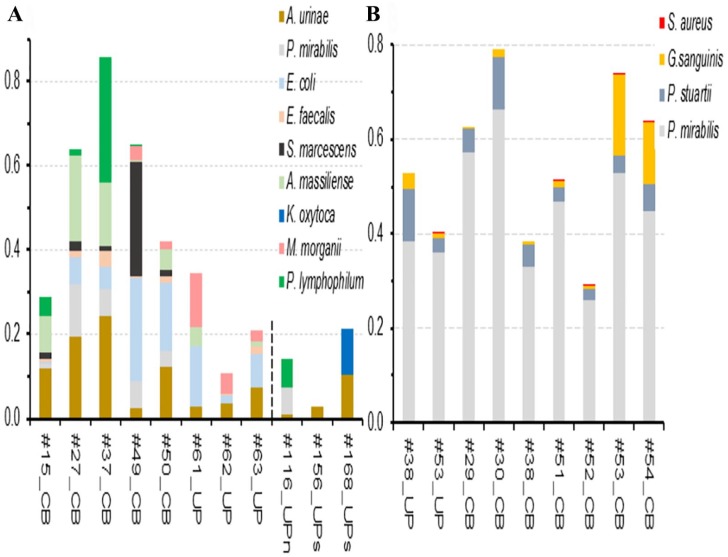
Relative quantities of polymicrobial proteomes in CB and UP samples from patients (A) P5 and (B) P6. The segmented bars are ordered from left to right according to the sequence of catheter collection time points. The time points of P5 and P6 were 2 and 3 weeks apart, respectively, thus indicating that the CBs persisted over several months. The colored segments of a bar represent the relative contribution of each microbial proteome to the entire sample’s proteome (including human proteins). Contributions of the latter (represented by the difference of 1 and the bar height) varied from 15% in #37_CB to 96% in #156_UP_s_. We estimate that the protein quantity is roughly equivalent to biomass contribution. Using color coding, the text on the right of the bar diagrams denotes the species represented by colored bar segments. A matching sample number for CB and UP samples in the plot (B) indicates specimen collection at the same timepoint. For comparative purposes, the graphic in (A) shows proteomic data from 3 unrelated cases of UTI with or without short-term catheterization (*Au* as one of the identified species; UPs: “s” for short term-catheterized; UPn: “n” for not catheterized). *Au* and *Gs* proteomes that were functionally interrogated in the following paragraphs are #27_CB, #37_CB, #168_UPs, #156_UPs, #53_UP, #53_CB, and #54_CB. CB indicates catheter biofilm; UP, urinary pellet; UTI, urinary tract infection.

Longitudinal surveys of microbial communities formed on bladder catheter surfaces from repeatedly catheterized patients with neurogenic bladders revealed that Aerococcaceae family members were part of persistent CBs, *Au* in 1 patient (P5) and *Gs* in another (P6). Their quantitative profiles derived from metaproteomic data are presented in Figure 1. 16S rRNA phylogenetic analyses confirmed the presence of *Aerococcus* and *Globicatella* at the genus level in the respective samples. The persistence of *Au* and *Gs* in the CB series supports the notion that these bacteria resist clearance when bladder catheters are replaced. The concept of bacterial dispersal from biofilms explains that all microbial species were identified in UP samples at equivalent time points (Figure 1). *Au* and *Gs* are not outcompeted by common uropathogens (eg, *Escherichia coli* or *Proteus mirabilis*) and Actinobacteria (eg, *Actinobaculum massiliense*) but share the microaerophilic biofilm niche. Previously, we found *Au* to be an infrequent cause of bacteriuria, observed in only 3 of 190 surveyed clinical cases.^20,24^

### Au and Gs strains isolated from microbial cultures

**Figure 2.**
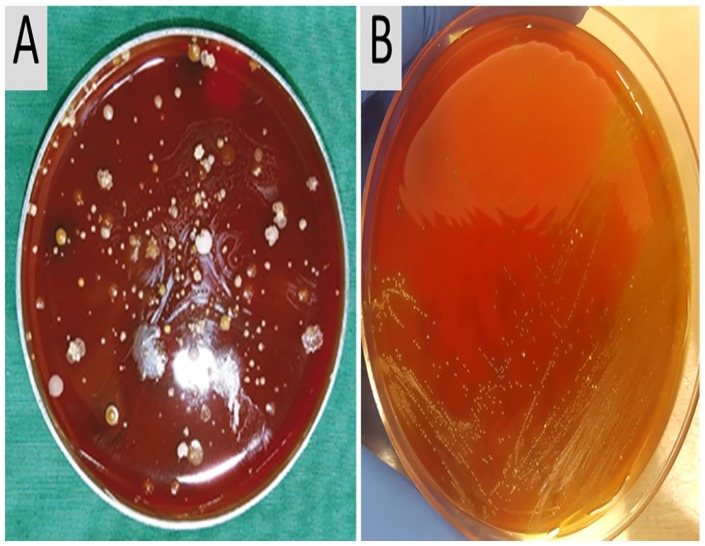
Anaerobically cultured bacteria from catheter biofilm extracts of P5 and P6. (A) Small grayish-white colonies on 5% sheep blood agar were identified as *Aerococcus urinae*. Eight other bacterial species were also identified from distinct agar colonies growing on the plate. (B) A *Globicatella sanguinis* strain from P6 (extract #53_CB) was grown aerobically on 5% sheep blood agar over 48 hours. The pinpoint-sized *Gs* colonies were α-hemolytic.

*Au* colonies were isolated from a catheter extract close to the collection timepoint of #63_UP. Figure 2 shows diverse microbial isolates from the sample’s anaerobic growth. A small white colony identified as *Aerococcus* by 16S rRNA sequencing was aerobically cultured in TSB liquid media, reaching an OD_600_ of 0.3. A CPP was isolated. A *Gs* strain from a catheter extract (#53_CB) was revived aerobically on sheep blood agar at 5% ambient CO_2_ and grew on this media in the form of pinpoint-sized, α-hemolytic colonies (Figure 2). A CCP was isolated from an entire agar plate re-grown from a single colony. The observed α-hemolysis may be due to peroxide production via a superoxide dismutase (CYJ72_08470), highly expressed in the in vitro-derived *Gs* proteome. Hemoglobin subunits accounted for 75% of non-microbial PSMs in this proteome, supporting the notion that sheep hemoglobin was solubilized by and became a nutrient source of *Gs*. In comparison, this was not observed for *Enterococcus faecalis* strains cultured similarly. Putative hemolysins (produced by β-hemolytic lactic acid bacteria) were not identified in the *Gs* proteome.

### Analysis of Au proteome in CCP and clinical samples in host proteome background

To our knowledge, this is the first report characterizing any *Aerococcus* proteome from either in vitro or in vivo environments. Three CB datasets, containing more than 450 *Au* proteins, were compared with 2 clinical *Au* datasets not linked to long-term catheterization (UP_S_ and UP_N_ samples in Figure 1) and 1 CCP dataset, all yielding at least 175 *Au* protein identifications. Human proteomes allowed inferences of innate immune responses against bacterial colonization. Symptoms were reported for the UTI datasets #116_UP_N_ and #168_UP_S_.^20,24^ The chronic bacterial colonization of catheters (P5 and P6) did not cause UTI symptoms. But the datasets revealed evidence of neutrophil infiltration irrespective of the symptomology: effector proteins such as myeloperoxidase, protein S100-A9, and α-defensin-1 (Table 1) were highly abundant. The inflammatory response can lead to tissue injury and exfoliation of urothelial and squamous epithelial cells. Hemoglobin and keratin-13, respectively, serve as indicators of such perturbations (Table 1). Neutrophil activities and epithelial exfoliation result in breakdown of proteins which, in addition to urinary salts, peptides, glucuronate-conjugated toxins, and pigments,^26^ serve as nutrient sources for bacteria in the inflamed urinary tract. In contrast, the bacterial proteome from CCPs reflects the response to a nutrient-rich growth media environment. A future goal is to adapt the nutrient and abiotic surface environments for *Au* and *Gs* with novel in vitro models simulating the catheter milieu.^27^

**Table 1.** Abundance of selected proteins associated with innate immunity and tissue injury.

Protein	116_UP_N_	168_UP_S_	156_UPs	UP (P5)	*var UPs (P5)	UPs (P6)	*var UPs (P6)
Myeloperoxidase	0.80	1.52	0.12	1.68	0.58	2.69	2.65
α-defensin-1	0.09	1.61	0.08	0.12	0.015	0.23	0.015
Calprotectin S100-A9	2.65	3.59	2.40	0.70	0.26	0.51	0.21
Uromodulin	6.97	8.97	1.93	2.67	2.48	3.07	0.95
Cytokeratin 13	1.73	0.52	3.50	1.68	1.21	0.07	0.002
Hemoglobin-α subunit	1.35	0.97	9.45	0.14	0.03	0.30	0.06

Abbreviations: LC-MS/MS, liquid chromatography coupled to tandem mass spectrometry; PSMs, peptide-spectral matches; UP, urinary pellet; CB, catheter biofilm.

Protein abundances are presented as PSMi/∑PSM (the sum of identified peptides for a given protein divided by the sum of all human PSMs profiled by LC-MS/MS in the respective dataset) averaged from 8 UP datasets for P5 and P6. Only UP but not CB data are included in the calculations because UP samples contain more human cellular matter in a microbially colonized urinary tract. *var: variance among 8 UP datasets for P5 and P6 each.

The entire bacterial proteomic dataset is provided in Supplemental File S2, with proteins as annotated in the genome of *Au* strain ACS-120-V-Col10a and with their quantities. LC-MS/MS raw files including human protein IDs were deposited in the PRoteomics IDEntifications (PRIDE) data repository via ProteomeXchange with the identifier PXD012047. Overall, 544 *Au* proteins were identified with at least 2 unique peptides, representing 32.4% of the in silico predicted proteome; 647 and 382 proteins (including those with 1 unique peptide) were identified from CB and UP_s_/UP_N_ data, respectively. Proteins that are predicted to interact with the host and/or contribute to bacterial fitness during colonization of catheters are listed in Table 2. Many of those were among the most abundant proteins in CB (in vivo) datasets.

**Table 2.** *Aerococcus urinae* proteins with potential roles in crosstalk with the host environment.

Gene locus^a^	Protein description^b^	Functional group or domain^c^	Put. role in interaction with host^d^	Predict location^e^	R (CB vs UPs)^f^	Q avg (CB)^g^
1626	Oligopeptide/nickel binding protein	ABC transporter su., MppA-type	Metal/heme/peptide uptake	CW; SP motif	4.7	0.0074
1619-1620	ABC transporter, ATP-binding proteins	ABC transporter su., MppA-type	Metal/heme/peptide uptake	CM	2.0; 12.3	0.0014; 0.0014
1621	ABC transporter, permease	ABC transporter	Metal/peptide uptake	CM	1.4	0.0015
1975	Receptor family ligand-binding protein	ABC transporter, HisP-type	Hydrophobic amino acid uptake	CW; SP motif	0.83	0.0032
1809	PTS, mannitol-specific IIC component	MtlA, component IIC	Mannitol uptake	CM	4.9	0.0028
1807	PEP-dependent sugar phosphotransferase system, EIIA 2	Kinase, component IIA2	Mannitol uptake	CM	2.3	0.0023
1806	Mannitol-1-phosphate 5-dehydrogenase	MtlC	Mannitol metabolic process	CY	1.3	0.0025
0913	d-xylulose 5-phosphate/d-fructose 6-phosphate phosphoketolase	Phosphoketolase	Xylulose/fructose metabolic process	CY	4.6	0.0339
0400	PfkB-type kinase	fructokinase, Scrk, PfkB type	Fructose/tagatose metabolic process	CY	11.8	0.0223
0304	Probable transaldolase Fsa	Transaldolase	Pentose-phosphate pathway	CY	>50	0.0091
0107	Transketolase	Tkt	Pentose-phosphate pathway	CY	28.5	0.0066
1602	Transketolase	Tkt, pyridin-binding domain	Pentose-phosphate pathway	CY	4.6	0.0089
0431	Uncharacterized protein, small protein		Pantothenate and CoA associated?	EX; SP motif	18.4	0.0021
0432	2-dehydropantoate 2-reductase	Oxidoreductive flavoprotein	Pantothenate and CoA synthesis	CY	1.26	0.0129
1406	NADH oxidase NoxE	O_2_-responsive signaling	Competence and virulence	CY	1.78	0.0079
1583	LPXTG-motif cell wall anchor domain protein	pilin subunit D1 domain	Host protein-binding, adhesion	LPATG CW anchor	7.3	0.0018
0550	LPXTG-motif cell wall anchor domain protein	mucin-binding domain, MucBP	Host protein-binding, adhesion	LPKTG CW anchor	1.58	0.0005
0479	Putative bacteriocin transport accessory protein	Thioredoxin accessory protein	Involved in bacterial competition, killing	CW; SP motif	6.03	0.0014
0915	Putative C protein alpha-antigen	Rib-α/Esp, Ig fold domains	Adhesion	CW; SP motif	<50	0.0032
0299-0300	Ferric iron ABC transporter binding protein	Ferric iron import	Response to iron sequestration	CW; SP motif	NA	0

Abbreviations: CB, catheter biofilm; CM, cell membrane; CW, cell wall; CY, cytosol; EX, exported; NADH, nicotinamide adenine dinucleotide; SP, signal peptide; UP, urinary pellet; Ig, immunoglobulin; MucBP, mucin-binding protein; CoA, coenzyme A.

a Gene locus (prefix HMPREF9243_).

b Description from the annotation in Gs genomes or from an ortholog.

c Functional role based on the entire sequence or a domain (data from UniProt: GO terms and/or InterPro references).

d Putative interactions with the host based on data from columns with b, c, and e footnotes.

e Predicted subcellular localization based on export signal sequence or cell wall immobilization. Subcellular localizations were predicted from transmembrane, secretion signal, and cell wall anchor motifs, as denoted in UniProt.

f Averaged abundance ratio for CB vs UPs datasets.

g Estimated relative protein quantity based on PSMi/∑PSM for the averaged CB datasets.

### Analysis of Gs proteome in CCP samples and clinical samples in host proteome background

To our knowledge, this is the first report characterizing a *Globicatella* proteome from either in vitro or in vivo environments. Two datasets with high *Gs* proteome coverage were compared with 1 UP dataset and 2 CCP datasets. We were interested in proteins differentially abundant in CB vs CCP datasets as proteins more abundant in CBs suggest a response to either human immune system activation or adaptation to in vivo nutrient sources. Merging proteins that matched sequences for the *Gs* strain UMB0514 and the *Globicatella* sp. HMSC072A10, the experimental proteome datasets are provided in Supplemental File S2. Overall, 627 proteins with at least 2 unique peptides, representing 26.3% of the in silico predicted proteome, were identified; 596 and 686 proteins (including those with 1 unique peptide) were derived from the CB and CCP datasets, respectively. Several Gs proteins with roles in either the host defense or indicative of tissue injury are listed in Table 3.

**Table 3.** *Globicatella sanguinis* proteins with potential roles in crosstalk with the host environment.

Gene locus^a^	Protein description^b^	Functional group or domain^c^	Put. role in interaction with host^d^	Predict location^e^	R (CB/CCP)^f^	Q avg (CB)^g^
09975	Zinc ABC transporter substrate-binding protein	ABC transporter, ZnuA-like	Metal ion uptake	CW; SP motif	25.4	0.0357
04450	Metal ion transporter	ABC transporter, ZnuA-like	Metal ion uptake	CW; SP motif	84.6	0.0109
*06785	Manganese ABC transporter ATP-binding Protein	ABC transporter, ATPase	Metal ion uptake	CM	4.4	0.0059
03785 03780	Oligopeptide ABC transporter substrate-binding protein	ABC transporter su., MppA-type	Metal/heme/peptide uptake	CW; SP motif	51.1; 1.7	0.04590.0205
02390	ABC transporter SBP	ABC transporter	Unknown substrate	CM	>50	0.0195
08170	Branched-chain amino acid ABC transporter SBP (LivJ)	ABC transporter, Leu/Ile/Val	Hydrophobic amino acid uptake	CW; SP motif	82.7	0.0052
*06620	PTS, mannitol-specific IIA component	MtlA	Mannitol uptake	CM	4.0	0.0040
*06610	PTS, mannitol-specific IIBC component	MltC	Mannitol uptake	CM	1.1	0.0001
*06625	Mannitol-1-phosphate 5-dehydrogenase	MtlD	Mannitol metabolic process	CY	17.9	0.0034
07330	BMP family ABC transporter substrate-binding protein	PnrA-like domain	Purine nucleotide uptake	CM	0.79	0.0067
08005	PTS mannose/fructose transporter subunit IID	ManZ	Mannose uptake	CM	>50	0.0020
08010	PTS mannose/fructose transporter subunit IIB	ManX	Mannose uptake	CM	>50	0.0020
*06835	PTS mannose/fructose transporter subunit IIC	ManY	Mannose uptake	CM	> 50	0.0017
02755	Putative MFS transporter superfamily	MFS transporter	Sugar/peptide/multi-drug transport	CM	3.7	0.0250
01545 01550	Citrate lyase α chain and β chain	Citrate lysate CitE, CitF	Anaerobic citrate metabolism	CY	15.4; 11.2	0.0197 0.0144
09665	Phosphonate ABC transporter SBP	ABC transporter for anions	Phosphonate uptake	CY	2.64	0.0108
03290	Peptidoglycan-binding protein, hydrolase	vWF/hemolysin domain homology	Peptidoglycan hydrolysis, adhesion, hemolysis	CW; SP motif	>50	0.0095
09120	Flavocytochrome c	Fumarate reductase	Electron transfer chain	EX; SP motif	>50	0.0084
02415	N-acetylneuraminate lyase	glycosylase	Sialic acid/glycan metabolism	CY	>50	0.0020
00745	CRISPR-associated endonuclease Cas9	RNA-guided endonuclease	Bacterial immune system	CY	>50	0.0008
09670	LPXTG-motif cell wall anchor domain protein	Ig-like fold domain	Host protein interaction, adhesion	IPNTG, CW anchor	0.84	0.0005
09805	Cupin domain-containing protein	RmlC-like jelly roll fold	Unknown	NA	4.8	0.0249
09290	Putative secreted protein	YSIRK motif	Unknown	YSIRK SP motif	4.0	0.0005

Abbreviations: CB indicates catheter biofilm; CM, cell membrane; CW, cell wall; CY, cytosol; EX, exported; PTS, phosphotransferase system; SP, signal peptide; UP, urinary pellet; vWF, von Willebrand factor; Ig, immunoglobulin.

a Gene locus of *Gs* strain UMB0514 (prefix CYJ72_).

b Description from the annotation in the genome of strain ACS-120-V-Col10a or from an ortholog.

c Functional role based on the entire sequence or a domain (data from UniProt: GO terms and/or InterPro references).

d Putative interactions with the host based on data from columns with b, c, and e footnotes.

e Predicted subcellular localization based on export signal sequence or cell wall immobilization.

f Averaged abundance ratio for CB vs CCP datasets.

g Estimated relative protein quantity based on PSMi/∑PSM for the averaged CB datasets. Subcellular localizations were predicted from transmembrane, secretion signal, and cell wall anchor motifs, as denoted in UniProt.

* Cases with prefix HMPREF2811_ (*Globicatella* sp. HMSC072A10 database).

### Putative Au and Gs protein interactions with host environment

*Au* expressed a putative C protein α-antigen containing a Rib-α/Esp cell adhesion motif. *Au* also expressed 2 LPXTG-motif proteins predicted to be cell wall-anchored and to interact via their N-terminal domains with the extracellular milieu, including human host factors. These proteins were more abundant in CBs than in CCPs (in vitro). *Gs* expressed 1 LPXTG-motif protein with an Ig-like fold predicted to mediate adhesion and a protein likely to be secreted because it has a YSIRK export motif. *Gs* also expressed a zinc ABC transporter lipoprotein, highly abundant in CBs (GS_09975). From here on, we use the term GS_ that replaces the prefixes CYJ72_ and HMPREF2811_ for ORF annotations. ZnuA-like protein orthologs for GS_09975 are EfaA in *E. faecalis* and MtsA in *Streptococcus pyogenes* (sequence identities of 41% and 38% with BlastP e-values 7e^−89^ and 2e^−82^, respectively). These cell surface lipoproteins have been associated with adhesion, virulence, transition metal ion (TMI) regulation,^28,29^ and TMI transport.^30^ Calprotectin S100-A9 (Table 1) and lactotransferrin (LTF) are human proteins that sequester zinc and iron, respectively. High abundance of these proteins may trigger induced expression of GS_09975 in vivo. Table 3 contains other *Gs* proteins with putative metal ion uptake functions. Surprisingly, potential TMI transporters were not identified in the *Au* proteome profiled from CBs. A potential cell wall–anchored protein with a high M_r_ and an IPNTG motif was identified but not abundant in vivo, while a secreted peptidoglycan-binding protein (GS_03290) was abundant in CBs (Table 3). GS_03290 does not have conserved motifs apart from a LysM domain suggesting a cell wall hydrolytic function. *Gs* expressed a CRISPR-associated endonuclease Cas9, predicted to protect the bacterium from the invasion of foreign DNA, in vivo. Other bacteria surveyed in the CBs (*P. mirabilis, P. stuartii*, and *S. aureus*) and their phages may trigger Cas9 expression. *P. mirabilis*, surveyed in the same samples (#53_CB and #54_CB), expressed phage shock proteins A and B (PspA and PspB), a putative phage replication protein (EP0005), and another putative phage protein (PMI0516). The interplay of other pathogens, phages, and the *Gs* CRISP-Cas9 system is an intriguing molecular research target for *Gs*-containing complex biofilms.

### Pathways used by Gs and Au to acquire nutrients in the CB milieu as inferred from proteomic data

**Figure 3.**
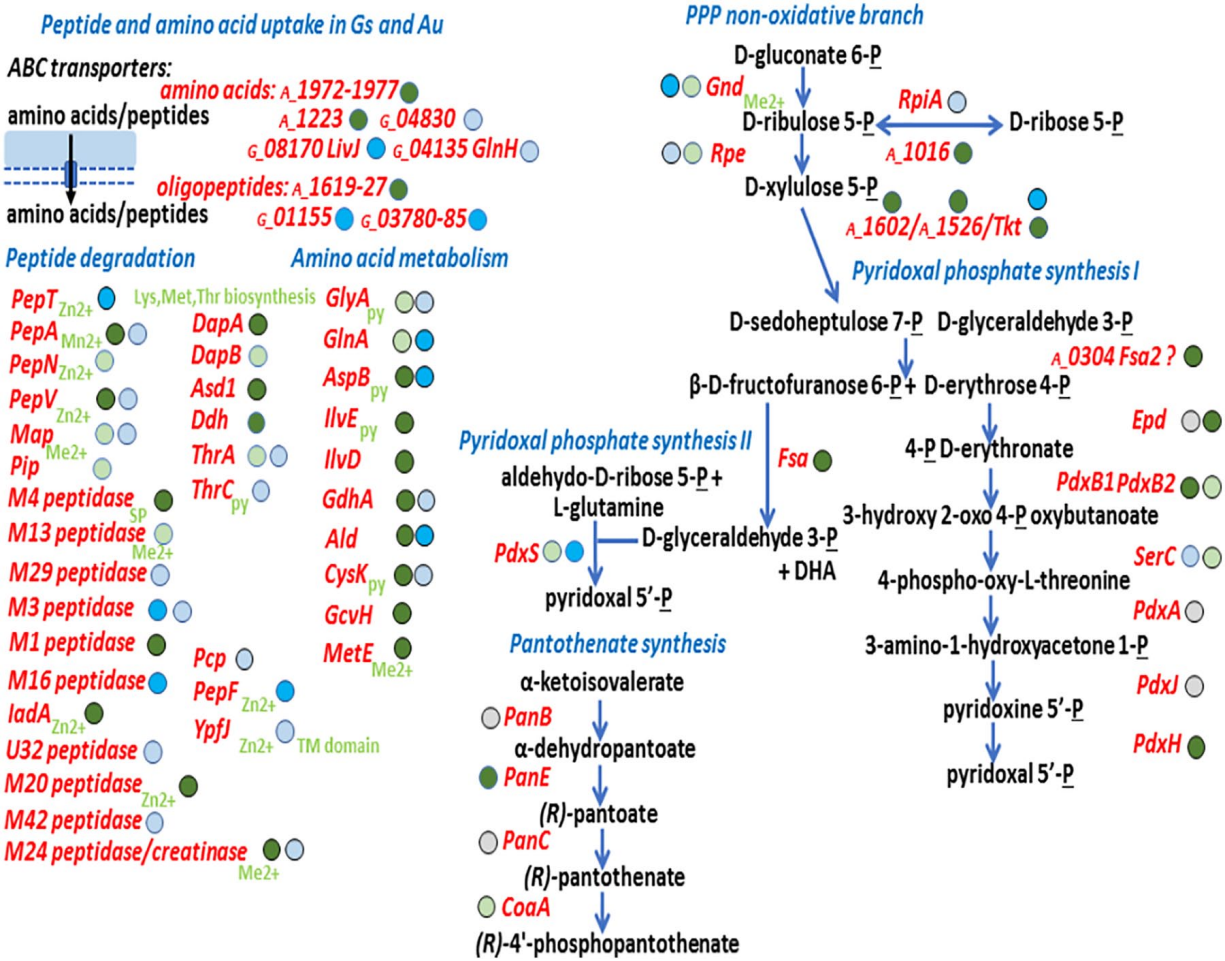
Peptide/amino acid transport and metabolism and cofactor synthesis in *Gs* and *Au* cells. The schematic representation contains protein names and gene loci (if protein short names were not provided for ORFs based on conserved sequences) in red. Each gene locus contains only the last 4 and 5 numbers of the *Au* and *Gs* gene accession terms, respectively. Details on the proteins (quantities and descriptions) are provided in datasets of the Supplemental File S2. Metabolite names are depicted in black. Blue arrows illustrate an enzymatic activity or pathway step, while black arrows indicate a transport process. The darker the color of the circle behind each protein name, the higher its average abundance in CB datasets. Blue: *Gs* proteins; green: *Au* proteins; gray: protein not detected in the proteomes. Cofactors, in light green script, are depicted next to enzymes where applicable: Me^2+^ (metal ion), Zn^2+^ (zinc), py (pyridoxal-5′-phosphate). DHA indicates dihydroxyacetone; “P,” phosphate; PPP, pentose-phosphate pathway; “?” indicates that a gene is predicted to catalyze an enzymatic step based on a domain with a predicted function or its gene neighborhood.

Among the abundant proteins in *Gs* and *Au* surveyed from the in vivo milieu were Mpp-type ABC transporters predicted to import di-/oligopeptides (GS_03780-GS_03785; AU_1619-AU_1626) and a few amino acid transporters (Tables 2 and 3, Figure 3). From here on, the term AU_ replaces the *Au* genome prefix HMPREF9243_. Recently, we also described the high in vivo abundance of 2 Mpp-type ABC transporters from *A. massiliense* in CBs, here a cohabitant in the CB series of P5.^18^ Apparently, oligopeptide import systems are key elements of nutritional fitness for lactic acid bacteria and Actinobacteria in CBs. Peptidases were more abundant in *Gs* and *Au* proteomes in CBs compared with in vitro proteomes. This included PepT, PepF, M3, and 1 M16 peptidases (*Gs*) and PepA, PepV, and the M1, M4, M20, and M24 peptidases (*Au*). Figure 3 also displays enzymes potentially contributing to the amino acid metabolism of *Au* and *Gs* in the CB milieu. Some of these enzymes are predicted to feature a pyridoxal-6′ phosphate (P6′P) cofactor. The P6′P biosynthesis pathway appeared to be active in *Au* (Figure 3). *Au* highly expressed 2-dehydropantoate 2-reductase (PanE), an enzyme part of the biosynthesis pathway of the cofactor (R)-4′-phosphopantothenate, in CBs. Pantothenate and CoA synthesis pathways have been proposed as antimicrobial drug targets.^31^ Several peptidases contain zinc cofactors (Figure 3). Predicted TMI transporters were not identified in the *Au* proteomes. In summary, both peptide and amino acid uptake and metabolism seem to be important functionalities enabling *Gs* and *Au* cells to generate the energy required for interactions with the host and growth in the catheterized human urinary tract.

### Sugar uptake, glycolytic and mixed acid fermentation pathway use by Au and Gs

**Figure 4.**
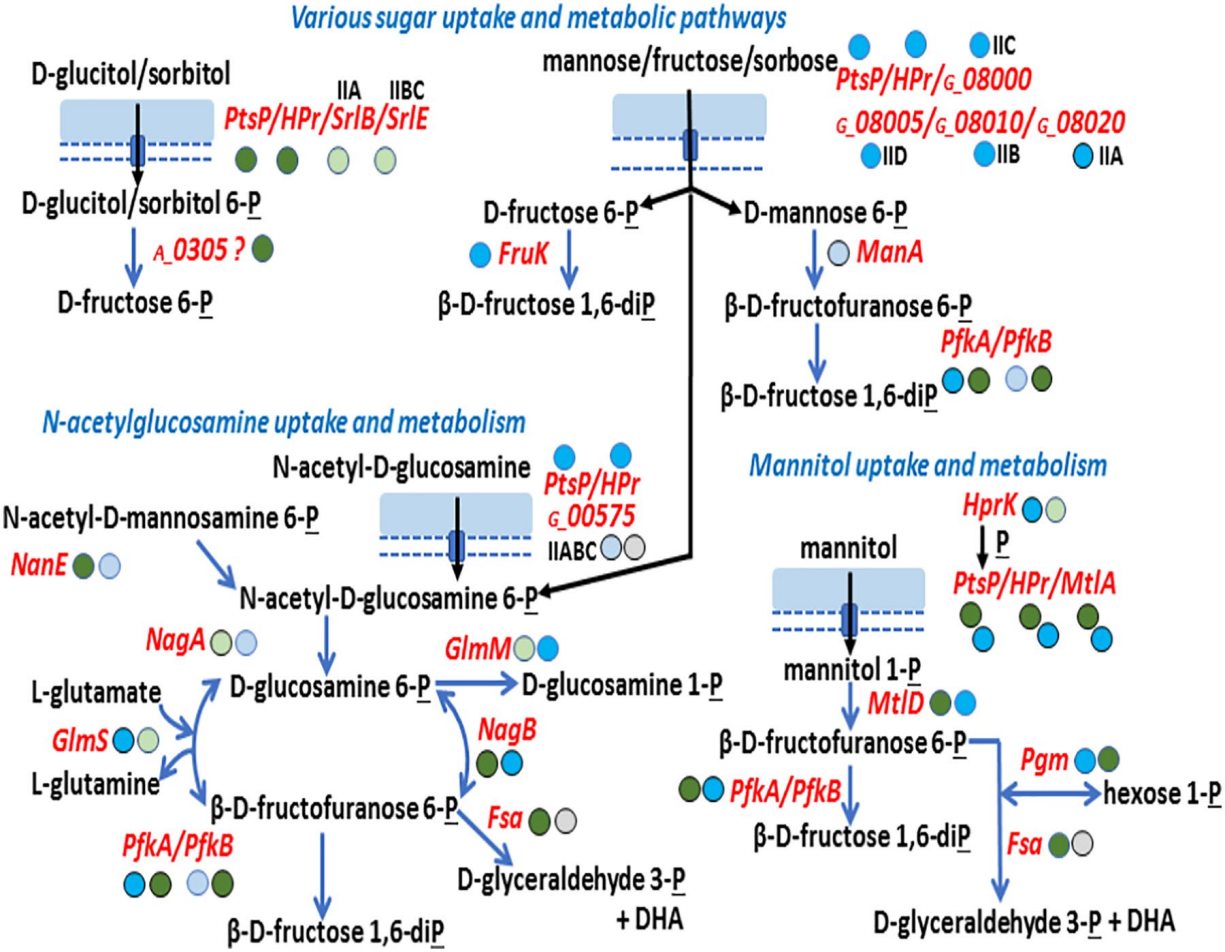
Inference of carbohydrate uptake and metabolism pathways used by *Aerococcus urinae* and *Globicatella sanguinis* in urethral catheter biofilms. The legend of Figure 3 already described most acronyms, symbols, and colors of circles that follow protein names/gene identifiers as well as the pathway connecting arrows used here. IIA, IIB, and IIC are terms generally used to define the subunits of PTS for ATP-dependent sugar import. ATP indicates adenosine tri-phosphate; PTS, phosphotransferase systems.

**Figure 5.**
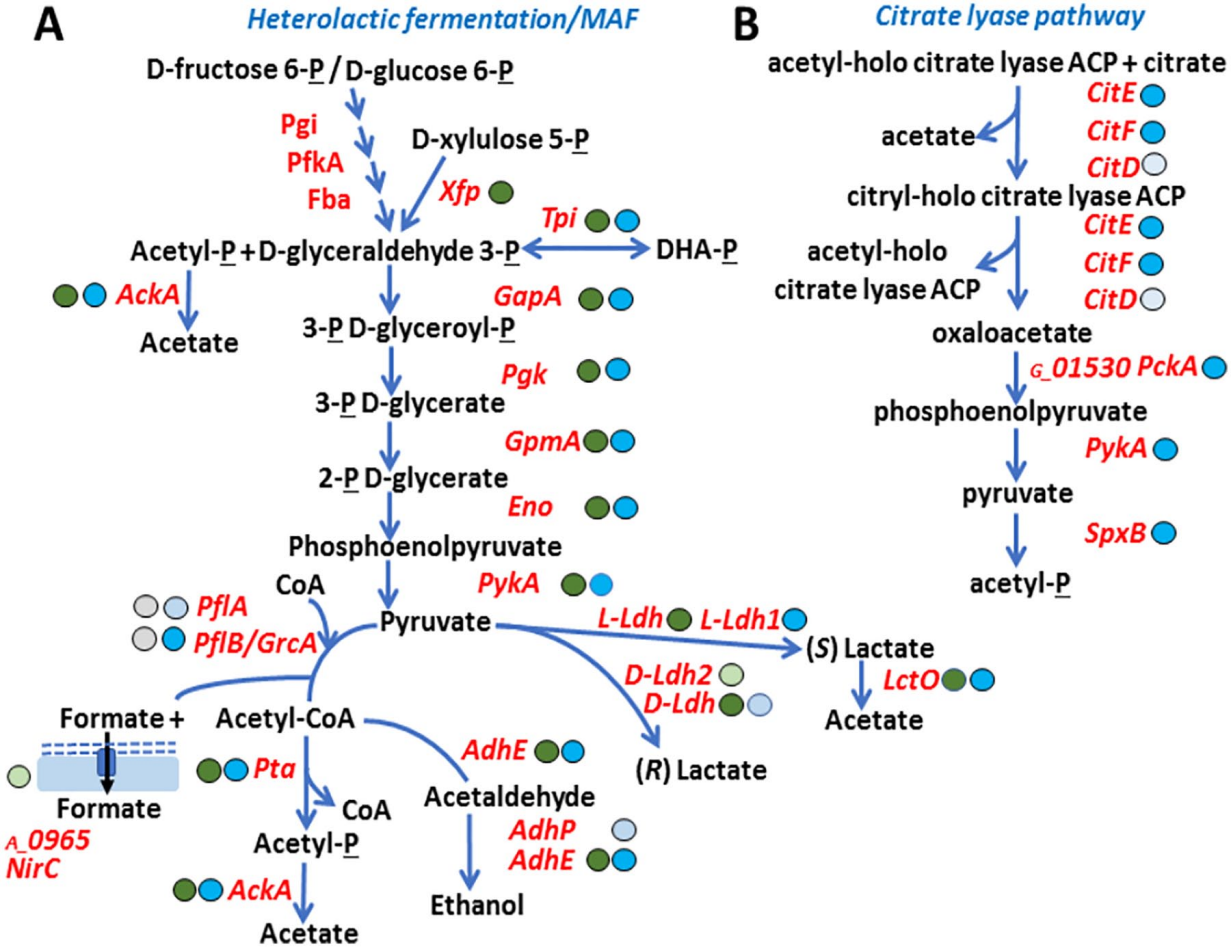
Active anaerobic energy metabolism pathways used by *Aerococcus urinae* and *Globicatella sanguinis* in urethral catheter biofilms. (A) Heterolactic fermentation pathways active in *Gs* and *Au*. (B) Citrate lyase pathway active in *Gs* only. The legend of Figure 3 describes most of the acronyms, symbols, and colors of circles that follow protein names/gene identifiers as well as the connecting arrows used here. The individual early glycolytic pathway steps catalyzed by Pgi, PfkA, and Fba (class II) are not shown in the schematic representation. These enzymes were highly abundant in the *Au* and *Gs* proteomes. MAF indicates mixed acid fermentation.

Proteomic data from CBs suggest that phosphotransferase system (PTS) is highly important for sugar uptake by these microbes in the urinary tract. The non-specific subunits of PTS, PstP and HPr, were abundant in the *Au* and *Gs* proteomes (both subunits in the top 20% based on PSM data). A glucitol/sorbitol PTS was highly expressed by *Au*. A mannose/fructose/sorbose PTS was abundant in *Gs*. Both organisms expressed mannitol uptake systems and enzymes to degrade this sugar and feed its phosphorylated derivatives into the glycolytic pathway (Figure 4). *Gs* highly expressed a PTS for N-acetylglucosamine uptake in the CB milieu. Both *Au* and *Gs* in vivo proteomes suggest an active metabolism of N-acetylated glucosamine and mannosamine, amino sugars that are components of many glycosylated proteins and glycosaminoglycans exposed on urothelial cell surfaces,^32^ as well as bacterial cell wall peptidoglycans. These macromolecules are accessible as nutrients for CB-associated bacteria. Expression profiles of 2 relevant enzymes (NagB and GlmS; Figure 4) were reported to change in opposite directions in *Streptococcus mutans*, thus influencing bacterial virulence.^33^ Among the most abundant proteins expressed by *Au* and *Gs* in vivo were enzymes of the glycolytic and mixed acid fermentation (MAF) pathways as illustrated in Figure 5A. The xylulose-5-phosphate degradation pathway catalyzed by the highly abundant enzyme Xfp in *Au* (Figure 5A) was absent in the *Gs* carbohydrate metabolism. This phosphoketolase is present in the genomes of many lactic bacteria. *Bifidobacterium lactis* Xfp was characterized as a dual-substrate, thiamine diphosphate-dependent enzyme important for bacterial fitness.^34^

### Differences in metabolic pathway use by Au vs Gs in the CB milieu

The citrate lyase pathway metabolizes citrate under anaerobic conditions and is well characterized in lactic acid bacteria, including the common uropathogen *E. faecalis*.^35,36^ Reducing equivalents (nicotinamide adenine dinucleotide phosphate (NADPH)) are not required for this pathway. We identified a complete representation of the citrate lyase pathway in the *Gs* strain but not in the *Au* strain (Figure 5B). The enzymes were much more abundant in the CB proteomes of *Gs* than in CCPs. A dedicated citrate transporter was not identified. Interestingly, citrate is a key inhibitor of urinary stones, and its utilization by lactic bacteria may enhance the risk of urinary stone formation.^37^ While the patients under investigation had no evidence of stones, inorganic salts had crystallized in several of the catheters derived from P6. This salt encrustation may result from urease-producing *P. mirabilis* that co-colonized the CBs from P6 and an increased urinary pH due to ammonia formed during the urea metabolism.^38^ The measured pH, however, was not above 7.5. Furthermore, the proteomic data suggested an active pathway for glycogen storage and metabolism in *Gs*, but not *Au*, in the in vivo milieu. Not all enzymes predicted to participate in the α-glucan degradation pathway of *Gs* were present in the proteome (Supplemental File S3). Pneumococcal glycogen metabolism was associated with this pathogen’s ability to mobilize glycogen as a nutrient source from lung epithelial cells.^39^

### Comparative analysis with uropathogenic Enterococci and Streptococci

While *Gs* and *Au* are considered rare uropathogens, the UTI epidemiology literature suggests that group D *Streptococci*, particularly *Enterococci*, are far more common causes of UTI and CAUTI.^40,41^ To some extent, this seems to be due to greater difficulties to culture *Au* and *Gs* resulting in underestimated numbers and misdiagnosis of clinical cases.^2^ Indeed, the attempts to isolate the *Au* strain of P5 from frozen cell extracts and to revive it on blood agar under aerobic conditions failed. In contrast, we frequently isolated an *E. faecalis* strain that co-colonized the P5 catheters. Due to the phylogenetic similarity, metabolic comparisons of *Au* and *Gs* strains from the CB milieu with that of *Enterococci* (*E. faecalis*) are appropriate. Like *Au* and *Gs*, the literature delineates that *E. faecalis* strains express a diversity of PTSs for the uptake of sugars and that the glycolytic, citrate lyase and MAF pathways are used to produce energy anaerobically.^42^ By adapting to a host milieu rich in amino sugars derived from glycosaminoglycans and mucosal cell surface glycoproteins, *E. faecalis* expresses transporters for oligopeptides and amino sugars as well as enzymes that feed these molecules into the peptidolysis and glycolytic pathways.^42^ Here, we demonstrate with a proteomic approach that *Au* and *Gs* express equivalent nutrient uptake systems and metabolism pathways. All 3 organisms have a highly active pyruvate dehydrogenase complex to degrade pyruvate and also metabolize pyruvate via other enzymatic pathways. According to our data, a pyruvate oxidase (SpxB) and a pyruvate-formate lyase (PflB/GrcA) are expressed by *Gs*, but not *Au*, in CBs. Datasets that showed evidence of *E. faecalis* co-colonization in CBs (in P5 and patients who we do not report on here) suggested a similarly high abundance for PflB/GrcA but not for a pyruvate oxidase in the *E. faecalis* proteome.

*Enterococci* and *Streptococci* produce a nicotinamide adenine dinucleotide (NADH) oxidase (Nox), which is thought to contribute to the regeneration of NAD+ to support glycolysis, and an NADH peroxidase (Npr), which is important to decompose H_2_O_2_ during aerobic growth and a likely virulence factor.^42-44^ Peroxide-producing enzymes (MPO, EPX) are generated in the CB milieu via the infiltrating activated granulocytes that generate oxidative stress. While we observed NADH oxidase orthologs, neither an NADH peroxidase nor an alkyl hydroperoxide reductase was expressed by *Au* and *Gs* in CBs. It remains to be shown how the 2 species degrade H_2_O_2_ and handle oxidative stress. *Au* and *Gs* expressed a superoxide dismutase to cope with oxidative stress in the CB milieu. The enzymes, a Cu/Zn type dismutase (*Au*) and an Fe/Mn-type dismutase (*Gs*), were highly abundant in the in vivo proteomes. We identified a few predicted surface-localized proteins that may have functional roles in the survival of *Au* and *Gs* in the host. The peptidoglycan-binding protein GS_03290 and a YSIRK secretion motif protein GS_09290 were increased in abundance in CBs compared with in vitro cultures of *Gs*, whereas 2 LPXTG motif proteins and a protein with a Rib-α/Esp adhesion domain were highly abundant in the *Au* proteomes derived from CBs. Whether these proteins are *Au* virulence factors needs to be established. In *E. faecalis*, the Rib-α/Esp adhesion protein Esp promotes primary attachment and biofilm formation on abiotic surfaces.^45^ While we characterized the proteomes and inferred metabolic capabilities of *Au* and *Gs* in the human urinary tract for the first time, our data do not clearly show that these gram-positive bacteria are opportunistic pathogens—or just bystanders that join a complex microbial community present in CBs that clearly recur in patients. Our data do not yield insights into the question as to whether prior colonization of catheters with known opportunistic pathogens such as *P. mirabilis, E. coli*, and *E. faecalis* is required for *Au* and *Gs* to cohabitate an existing biofilm. This study sets the stage for further genetic and biochemical investigations to shed more light on the role of *Au* and *Gs* in urethral CBs.

## Concluding Remarks

We demonstrate that 2 species of the Aerococcaceae family, *Au* and *Gs*, can colonize urethral catheters recurrently. A phylogenetic relative, *E. faecalis*, is far better characterized as a pathogen adapted to the CB niche.^40,42,45,46^ Unlike for *E. faecalis*, virulence factors facilitating the adhesion to host proteins and cell surfaces and extracellular proteases have not been characterized for *Au* or *Gs*. While our data offer a few virulence factor candidates based on motifs and abundances in the host milieu, we argue that a continuum from high to low virulence exists for a biological niche susceptible to infection, and that *Au* and *Gs* are on the low virulence end and thus thrive more in a polymicrobial environment where they hide from the hostile human immune system.

## Supplemental Material

Suppl_Materials_File_S1 – Supplemental material for Aerococcus urinae and Globicatella sanguinis Persist in Polymicrobial Urethral Catheter Biofilms Examined in Longitudinal Profiles at the Proteomic Level Click here for additional data file. Supplemental material, Suppl_Materials_File_S1 for Aerococcus urinae and Globicatella sanguinis Persist in Polymicrobial Urethral Catheter Biofilms Examined in Longitudinal Profiles at the Proteomic Level by Yanbao Yu, Tamara Tsitrin, Shiferaw Bekele, Vishal Thovarai, Manolito G Torralba, Harinder Singh, Randall Wolcott, Sebastian N Doerfert, Maria V Sizova, Slava S Epstein and Rembert Pieper in Biochemistry Insights

## Supplemental Material

Suppl_Materials_File_S2 – Supplemental material for Aerococcus urinae and Globicatella sanguinis Persist in Polymicrobial Urethral Catheter Biofilms Examined in Longitudinal Profiles at the Proteomic Level Click here for additional data file. Supplemental material, Suppl_Materials_File_S2 for Aerococcus urinae and Globicatella sanguinis Persist in Polymicrobial Urethral Catheter Biofilms Examined in Longitudinal Profiles at the Proteomic Level by Yanbao Yu, Tamara Tsitrin, Shiferaw Bekele, Vishal Thovarai, Manolito G Torralba, Harinder Singh, Randall Wolcott, Sebastian N Doerfert, Maria V Sizova, Slava S Epstein and Rembert Pieper in Biochemistry Insights

## Supplemental Material

Suppl_Materials_File_S3 – Supplemental material for Aerococcus urinae and Globicatella sanguinis Persist in Polymicrobial Urethral Catheter Biofilms Examined in Longitudinal Profiles at the Proteomic Level Click here for additional data file. Supplemental material, Suppl_Materials_File_S3 for Aerococcus urinae and Globicatella sanguinis Persist in Polymicrobial Urethral Catheter Biofilms Examined in Longitudinal Profiles at the Proteomic Level by Yanbao Yu, Tamara Tsitrin, Shiferaw Bekele, Vishal Thovarai, Manolito G Torralba, Harinder Singh, Randall Wolcott, Sebastian N Doerfert, Maria V Sizova, Slava S Epstein and Rembert Pieper in Biochemistry Insights
