# Supplementary material for: Aerococcus urinae and Globicatella sanguinis Persist in Polymicrobial Urethral Catheter Biofilms Examined in Longitudinal Profiles at the Proteomic Level
Source: Biochem Insights. 2019 Sep 19;12:1178626419875089. doi: 10.1177/1178626419875089 (PMC6753514; doi:10.1177/1178626419875089)

Figure S1 (Suppl. File S3).

Pathways for glycogen biosynthesis and α-glucan degradation inferred to be active in the catheter biofilm milieu based on the expression of contributing *G. sanguinis* enzymes. The schematic contains protein names and gene loci (in five numbers; the prefix HMPREF2811_ from the *Globicatella sp.* HMSC072A10 database) if protein short names were not provided for ORF) in red. Detailed descriptions for the proteins (quantities and descriptions) are provided in datasets of the Suppl. File S2. Metabolite names are depicted in black. Blue arrows illustrate an enzymatic activity or pathway step, while black arrows indicate a transport process. The darker blue the color of the circle behind a protein name, the higher its average abundance in CB datasets; proteins in grey: part of the pathway but not detected in the proteomes. Cofactors in light green script: py - pyridoxal-5'-phosphate; 'P': phosphate; '?': indicates that a gene is predicted to catalyze an enzymatic step based on a domain with a predicted function.

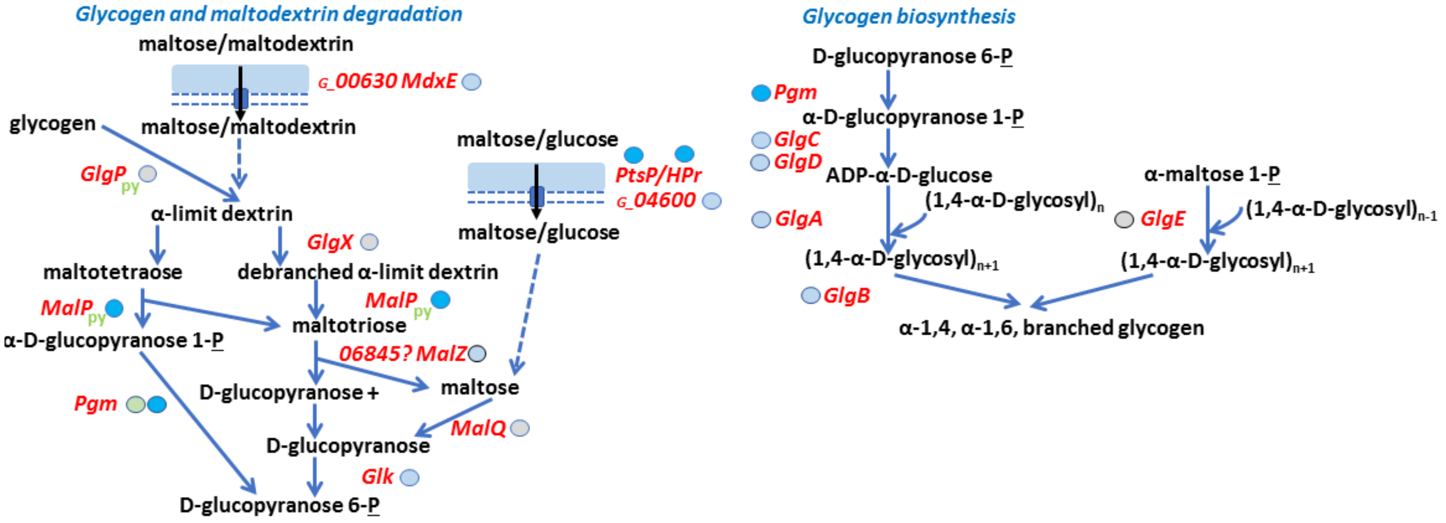

Supplement: Suppl_Materials_File_S3 – Supplemental material for Aerococcus urinae and Globicatella sanguinis Persist in Polymicrobial Urethral Catheter Biofilms Examined in Longitudinal Profiles at the Proteomic Level [file Suppl_Materials_File_S3.pdf]
